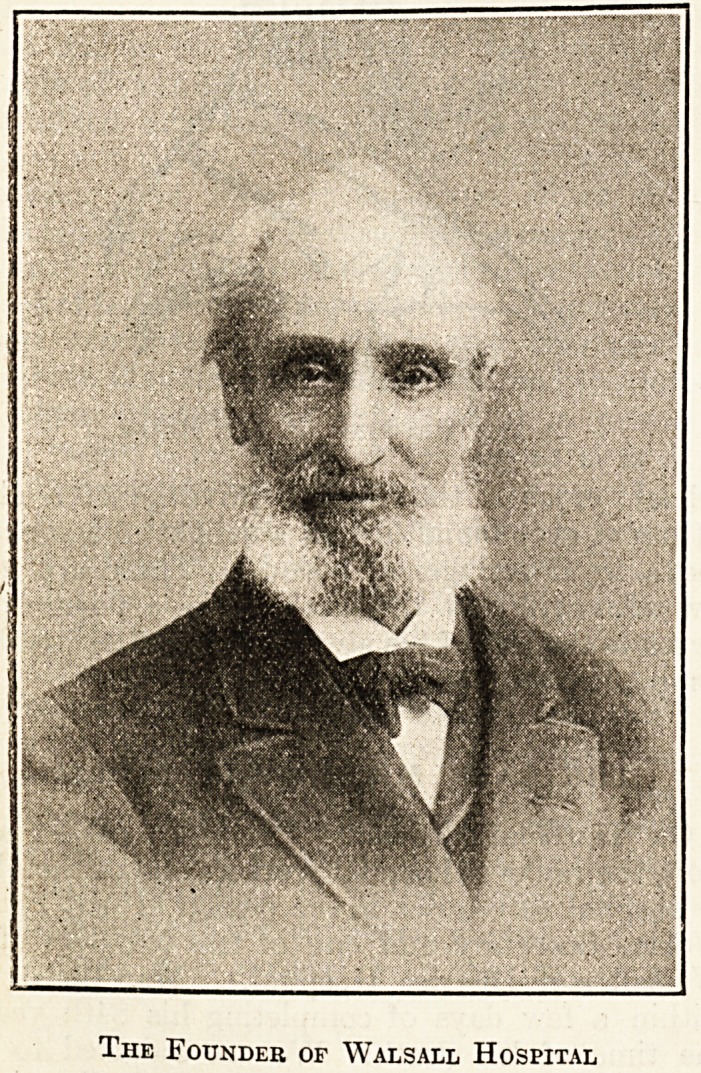# The Late Mr. Arthur Reade and the Late Mr. Samuel Welsh

**Published:** 1912-02-03

**Authors:** 


### TWO USEFUL CAREERS.

### The late Mr. Arthur Reade and the late Mr. Samuel Welsh.

The intelligence of the death of Arthur Edward Reade, formerly and for many years the zealous and able secretary of Charing Cross Hospital, has caused profound sorrow and regret to his many friends in the hospital world. For some little while the state of his health has not been quite so satisfactory as his friends could have desired, but it was only very shortly before his death that there was any real cause for anxiety. Shortly after Christmas he suffered from a slight attack of influenza, from which he appeared to have recovered, and he was out of doors so recently as ten days before his death, which occurred on Friday afternoon last, at his residence in Streatham, from pleurisy and pneumonia. A personal friend of the late Mr. Reade has sent us the following memoir:—

### A Personal Tribute.

Arthur Reade belonged to an old Oxfordshire family, having been the youngest son of the late Edward Anderson Reade, C.B., of Ipsden in that county. Born in the year 1848, he was educated at Tonbridge and Haileybury, whence he proceeded to India in the Government telegraph service. Returning home on furlough in due course, he was sc fortunate as to contract a happy marriage, an event which altered the whole course of his career, as it led to his remaining permanently in England. After a while, turning his attention to hospital work, he served for a short time as secretary to the Hospital for Paralysis and Epilepsy, a post which he relinquished on his appointment in the year 1882 to the more important and responsible position of secretary to Charing Cross Hospital. Here the best years of his life were passed, much to the advantage of the institution which he so faithfully served for twenty-four years. Upon his retirement in the summer of 1906 he still continued to lead an active life and was always foremost to undertake any service and to perform any act of kindness which might be of benefit to his friends and neighbours. The local dispensary was glad to secure his services as honorary secretary. At the time when there was much poverty and distress in consequence of lack of employment he readily undertook the task of organising measures for the relief of the unemployed, and it is a striking testimony to his great tact and sympathy that his efforts were valued and appreciated alike by those who provided the means for helping the distressed and by those for whose sake he laboured so strenuously.

Upon the conclusion of the litigation which, as will be remembered, took place not very long ago in connection with the Weir Hospital bequest, the trustees of that charity co-opted Arthur Reade as one of their number, being glad to avail themselves of his knowledge of hospital work. More recently still he consented to become one of the guardians of the poor for the Wandsworth union, the municipal borough in which he resided.

Notwithstanding all these varied occupations Arthur Reade found time for other and lighter pursuits. A sportsman in the best sense of the word, he took a keen interest in cricket and other games. This is perhaps one of the reasons why children were so peculiarly attracted to him, and it was no uncommon occurrence to find him surrounded by groups of boys and girls helping them to organise their games.

Those who, in addition to their acquaintance with Arthur Reade as an able and efficient hospital

A Pen Drawing of Mr. Reade.

official, enjoyed the further privilege of a closer intimacy, ever found in him a delightful companion and a loyal and genial friend. There are not a few who will find the world to be a poorer place owing to the loss of his always kind and sympathetic companionship.

### THE LATE MR. SAMUEL WELSH.

On Thursday, January 18, there passed away from the ranks of hospital workers one of the oldest of hospital secretaries in the person of Mr. Samuel Welsh, Secretary and one of the founders of the Walsall and District Hospital. Mr. Welsh was within a few days of completing his 84th year at the time of his death. We are indebted to Mr. Harold Wigg, co-secretary of the Walsall and District Hospital, for the following memoir:—

A Dumfriesshire man by birth, he came from Middlesbrough to Walsall nearly sixty years ago as a journalist, where, as editor of the *Free Press* for a large number of years, he associated himself with many important movements in the town, and in the course of his advocacy, both through the Press and on the platform, of useful reforms, he won for himself much admiration and respect.

His great work, the work indeed which was the one absorbing interest of his life, was the foundation and maintenance of the Walsall Hospital. Through his advocacy and untiring labours at a time when the need for such an institution seemed to him most pressing, the Walsall Cottage Hospital was opened in October 1863. Mr. Welsh brought his idea from Middlesbrough, where, during a short visit as a journalist, he became interested in the establishment of the first hospital instituted on the "Cottage" system.

In two small houses in Bridge Street, the work of the Walsall Hospital was commenced under the superintendence of Sister Mary, of Middlesbrough. Two years later Sister Mary was succeeded by Sister Dora, whose work is familiar to many hospital workers, and during whose lifetime the hospital acquired a high reputation.

The claims of the hospital were ever the subject of Mr. Welsh's earnest pleading, and Walsall owes him a debt of gratitude for his share in establishing and administering an institution which has done so much for the town, and is carrying on increasingly useful work.

Up to the time of his retirement from journalism, some twenty years ago, Mr. Welsh had acted as honorary secretary of the hospital; he then accepted the position of secretary, and devoted his whole energies to the work. In 1907 he was presented by his many admirers with a handsome cheque, as a recognition of the long and useful

The Founder of Walsall Hospital services he had rendered; since that date, though nominally continuing as secretary, he was, through ill-health, unable to take any active part in the work.

Mr. Welsh possessed an extremely wide knowledge of matters associated with hospital work; he was much interested in the provision of convalescent homes, and rendered some assistance in that direction. His latest desire, and one which he was not destined to see fulfilled, was the establishment of homes for rest for nurses, and he attempted, unsuccessfully, to get his idea carried into effect as a memorial to the late Miss Florence Nightingale. He was endowed with an attractive and interesting personality; he had read widely in many subjects, and possessed a retentive memory to the last. Almost up to the day of his death his energies were spent in humanitarian service.

## Figures and Tables

**Figure f1:**
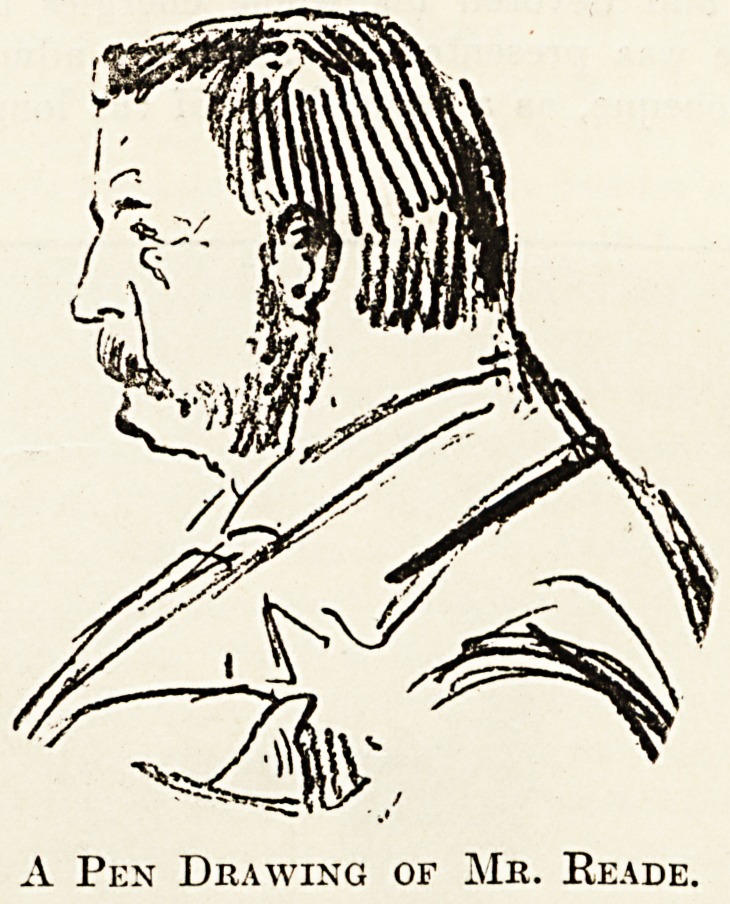


**Figure f2:**